# A Treatise on the Diseases of Newly-Born Infants, and of Their Diseases during Lactation

**Published:** 1834-04-01

**Authors:** 


*A Treatise on the Diseases of Newly-born Infants, and of their Diseases during Lactation; founded on Clinical Observations, and on Pathological Anatomy, made at the Foundling Hospital, Paris.* By C. M. BILLARD, Doctor of Medicine of the Faculty of Paris, and Member of other Learned Societies. *Second Edition, enlarged by a Medico-legal Memoir on the Vitality of the Fœtus, with Notes, and a Notice of the Life and Works of the Author, by* M. OLLIVIER (D'ANGERS.)—Paris. Pp. 720.

THE diseases of children have received less attention than they deserve, which is more surprising and to be regretted, considering their importance and the interesting character of the sufferers. The fact cannot be concealed, and assuredly not excused. If an objector should be found to this proposition, we challenge the production of any work comprehending the peculiarities of infant health and disease, on which the practitioner can rely for sure guidance, when embarrassed by their apparent anomalies. *Anomalous* they may be called, because, although they are subject to the same general laws as in adults, the ever-varying conditions of infancy give them a new character, and compel a corresponding variation in the treatment. It were useless to insist upon propositions all but self-evident: frequently has the humane practitioner looked back with sorrow and regret upon that period of his career, when, not having by time acquired experience in treating the diseases of children, his failures bore a painful and humiliating contrast to the success of his riper knowledge. Indolence or culpable indifference may attempt to screen itself under the excuse of the difficulty of a diagnosis formed from data rendered uncertain by the inaptitude of the patient to give an account of the origin, progress, and symptoms of disease. Often as this disqualification is insisted on, we have never assented to it: on the contrary, we hold fast by a belief, a conviction directly the reverse; for it can frequently be shewn that the diseases of children admit of a much more accurate distinction than the same in adults. Suppose a child breathing with frightful rapidity, seventy in a minute, pulse 140 or 160, skin hot, eyes suffused, features swollen and anxious, cough frequent, restlessness incessant; is a single question necessary to determine that the disease is pneumonia, which will almost certainly prove fatal? Cerebral affections are also much more distinctly and strongly marked in infancy. The simple habits of children also exempt them from many diseases of adolescence. Again, the latency of some functions only to be developed by puberty prevents many complications which embarrass in the diseases of maturity. It is, however, needless to multiply examples, though to do so would be easy. The infant sick are also more manageable; for the recusancy of children is controllable, while contention with the ignorance or obstinacy of grown persons is often as useless as it is disgusting. In another number of this Journal we have put upon record our opinion, that our defective knowledge on the subject before us was mainly attributable to the ignorance or supineness of the teachers of midwifery. Take a fair sample of the whole class.

The lecturer, for whose opinion and prelections we have the highest respect, whose judgment and opportunities place him, in our estimation, immeasurably in the van of his competitors, was in the habit of contenting himself with alluding to "hydrocephalus" (the name of a species, although always employed for the genus in these days,) in the tail of one lecture. His description of the symptoms was as brief as his directions for the treatment: "Fever—constipation—vomiting—green stools—convulsions—squinting—death. Treatment: Leeches—venesection—cold applications to the head—blisters to the nape of the neck—warm bath—calomel inside, and ungt. hydrarg. all over out,—winding up with fœtid enemata." And, by way of fortifying these directions, it was added, "Gentlemen: the reason of our too frequent failure in the treatment of this disease is, that we do not bleed sufficiently. If you treat an inflamed knee, do you tamper with three or four, or even a dozen leeches? No! to be sure, you apply twenty or thirty, or a hundred: and, gentlemen, what is the corollary? Why, if an inflamed knee require so many leeches, what must an inflamed brain do? If it require many for the member for genuflexion, it must require more for the organ of thought."

Something like this was the peroration of a lecture by at least as able a teacher, and as skilful a practitioner, as any in these islands, ever excepting on the theme in hand.

An extensive acquaintance with our brethren justifies the assertion, that the most successful are those whose knowledge is the result of a large experience, rather than the inculcation of sound principles in connexion with the subject of infantile diseases: their knowledge is therefore almost purely empirical; and, as it was conceived in ignorance, its first fruits must have been bitter. No humane man can do otherwise than lament the want of a guide in this wilderness, until familiarity with the oft-travelled track shall enable him to journey alone. This is written advisedly, and with the full conviction of the great merits of Underwood, Burns, and Dewees, whose works are indispensable to a good intelligence of these maladies.

In pathology it is no longer disputed that the French have almost incalculable advantages over the English, and in no instance is this advantage more conspicuous than the present. Even our national self-love cannot conceal the fact demonstrable in every line of this treatise, that M. Billard has alone distanced all the English pathologists who have devoted their best time to the morbid anatomy of the diseases of children.

We have no motive for selecting any one portion of the work in preference to another for especial notice, because the details are desultory, and not of the systematising kind, so frequently distinguishing the labours of French pathologists. The orthodoxy of ignorance may reject some of M. Billard's views as visionary, and there may be some who, glorying in the description of "practical men," will reject his theories, when they occur, as refined and unsubstantial subtleties. To these it were useless to commend the present volume.

Clinical and pathological reports, form a large portion of the book, and constitute its chief value. M. Billard's reasonings are never inconsequent, and but rarely savouring of the imaginative character of French theorists: he instructs more by the faithful and vivid portraiture of symptoms and morbid appearances, than by his hypotheses on their producing causes.

The following case is an interesting one:

"Marie Dumé, aged six days, of rather a strong constitution, entered the Infirmary on the 13th October. The countenance was flushed, but the limbs and trunk were slightly jaundiced; she cried but little, and slept quietly; the mucous membrane of the mouth was in the natural state, but the tongue was red at the tip and edges. On the 15th October the jaundice was less intense, and a rather copious diarrhœa followed; the gums were swollen, without being red; the child cried much, and was paler. This state of things continued till the 20th October, when the purging increased, and the child became very pale; the upper gums were swollen, and of a livid red colour. On the 25th all these symptoms increased; the child's cry became feeble, the pulse gentle and slow; but there was no cessation of the diarrhœa, and it died in the night.

"On examination *post-mortem*, the upper gum on the right side presented a livid tumefaction with fluctuation: in the three sockets of the first teeth there was an effusion of blackish fluid blood. The incisors, and that part of the germ which is not ossified, floated free and detached in the effused blood which distended the tumour; the bony cavities of the teeth were flabby, red, and as it were infused in the fluid. The surrounding soft parts began to detach themselves from the alveolar edge. The rest of the mouth was healthy. At the cardiac orifice some red striæ crossed the surface of the stomach; the mucous membrane at the end of the duodenum was thick and tumefied; near the valvula coli were red and tumified follicles. The liver was gorged with blood ; the bile was abundant, viscid, and of a pale green. The lungs, heart, and brain, were healthy."

He proceeds to observe, that the case offers two remarkable circumstances for consideration: one, that symptoms having their seat in the teeth and their germs can occur in the first days of life; the other, that hemorrhages occur, as in the present example, from congestion of those parts, which is so common in infants at their birth.

While admiring the clear and luminous description, English practitioners will marvel at the obvious inertness of the treatment, often the characteristic of French therapeutics, as uncalled-for energy too frequently distinguishes our own.

We will now gratify our readers with a larger extract from this useful work.

"§ 1. *Congestions.* Passive congestion of the cerebral and spinal system is very common in new-born infants. It results from the high vascularity and slow circulation of the parts, and the influence of breathing upon their circulation. Protracted labour, the force necessarily used in particular cases of manual delivery, the difficulty with which respiration is established, the sudden change which takes place in the circulation of the infant, are additional and frequent causes of this affection; and it may exist in any degree from simple injection of the meninges to true apoplexy.

"Various degrees of cerebral congestion are comprehended under the general term of apoplexy of new-born infants; nor must we expect, in the majority of those who die apoplectic, to find that peculiar extravasation of blood, or any circumscribed cerebral hæmorrhage, which constitutes the malady of the same name in adults. We will now proceed to review the different lesions which are found in connexion with this disease.

"Injection of the meninges, of the chord, and of the brain, is so common in the new-born infant, that I am disposed to consider it rather as a natural state of parts than as a disease. It is found in the greater number of instances. Vascular injection, and even extravasation of blood at the inferior and posterior extremity of the spine, are very frequent. I have often observed it, though there had been no appreciable symptoms of such an affection during life.

"If the injection be excessive, a bloody exudation quickly takes place on the surfaces of the meninges, and the blood thus exhaled, commonly more or less coagulated, compresses the brain or the spinal marrow, and gives rise to the state of stupor or depression which characterizes apoplexy. This hemorrhage, external to the cerebral mass, is almost constantly met with in infants who are said to have died of apoplexy. It is this which M. Serres denominates meningeal apoplexy, and which he attributes to the rupture of some of the vascular branches which are distributed on the surface of the brain.

"Injection of the pulp of the brain is also not uncommon. It appears in the form of minute red points or dots, occasionally tinges the substance of the organ with a somewhat deep red colour, and is chiefly found on the lateral parts of the corpora striata and the optic thalami. It is here, in fact, that the vessels of the brain are the most abundant, and that cerebral hemorrhages and inflammations are most frequent at every period of life. This point has been incontestably proved by the writings of Morgagni, and the recent researches of MM. Lallemand and Bouillaud.

"Cerebral hemorrhage is found sometimes, but rarely; I have met with but a single example of it. The infant had died on the third day after birth, and with the ordinary symptoms of apoplexy: upon opening the body, a clot of blood was found in the substance of the left hemisphere, by the side of the corpus striatum. There was apparently no cyst; the cerebral substance was only a little softened in the neighbourhood of the clot, which was about an inch long and half an inch broad.*

"§ II. *Softening without Inflammation.* This is a lesion peculiar to the brain of new-born infants, and is evidently the result of the congestions of this organ. I speak of a species of local or general softening, which, far from presenting the characters of inflammation, offers, on the contrary, all the appearances of the decomposition, I might almost say putrefaction, of the organ. I will begin by relating an instance of this.

"Alexis Lonatt, aged three days, was placed in the Infirmary on the 18th of May. He was affected with general induration of the cellular tissue; the integuments were of a violet red colour all over the body; his cry was feeble and uneasy, and occasionally piercing; there was very slight resonance of the chest upon percussion; he was moreover affected with a very abundant diarrhœa, and the stools were green; the pulsations of the heart were rapid, but extremely feeble. No change took place during the next few

* "A fact observed by the younger Bérard proves that cerebral hemorrhage may take place in the fœtus in utero; so that apoplexy should be enumerated among those diseases of which a child may perish before its birth, and which may cause abortion. The fœtus in which M. Bérard observed this remarkable lesion was aged eight months and a half; the clot, of the size of a nut, was lodged in the substance of the brain."—*Compte rendu de la Société Anatomique, pour l'année* 1828.

days, and he died on the 21st of May. Upon a post-mortem examination, the organs of digestion were found much loaded with blood through their whole extent. The liver was gorged with dark fluid blood, its tissue hardened, and of a brown slate colour. The lungs were flaccid, blackish, but little distended with air, and the posterior part gorged with blood. The fœtal openings were still permeable, the meninges much injected; the cerebral mass reduced to a semifluid pulp, which oozed from every incision through the arachnoid, and smelt strongly of sulphuretted hydrogen. This softening extended to the lateral ventricles, where extravasated blood was found in considerable quantity. The rest of the brain was softened, and of a violet colour, but it was far from being of the semifluid consistence of the portion of the hemispheres above the ventricles.

We may suppose "that this general disorganization of the pulp of the brain was the result of its contact and mixture with the blood extravasated in the ventricles, and infiltrated into the substance of the brain itself. This softening, remarkable for its resemblance in colour to the lees of wine, and its strong odour of sulphuretted hydrogen, is often the result of the mixture of blood with the substance of the brain, for a cerebral hemorrhage almost invariably accompanies it; but this hemorrhage, when it is recent, may exist alone, the softening of the pulp of the brain not having yet taken place: still we observe, either at the upper part of the hemispheres, or externally to the corpora striata, portions of the brain which are beginning to soften, and have already the odour peculiar to this disorganization. On the other hand, I am inclined to believe that the cerebral softening may precede the hemorrhage, and may even be the occasion of it, for I have found it several times without the extravasation of blood.

"The softening of which I speak sometimes only exists in one lobe, at other times in both; very frequently the whole mass of the brain is thus destroyed; and, when opening the cranium, nothing is found but a semifluid mass, of a dark colour, and mixed with a great number of bloody clots and pulpy shreds. It is a very remarkable fact, that the meninges never partake of this disorganization, and that, notwithstanding such extensive destruction of the encephalon, the infants still exist for several days. It is true that, according to the vulgar phrase, they have scarcely a breath of life in them, but still they breathe, cry, and can suck: this is owing to the circumstance that the disorganization generally stops short of the medulla oblongata, which remains untouched, and presides, with the spinal marrow, over the vital functions, which it supports for a short time.

"I have often found this softening in new-born infants that had died immediately after birth, which disposes me to believe that it had occurred during the existence of the child in the womb.

"When the medulla oblongata and the spinal marrow are thus softened, the child displays a much inferior degree of vital energy; its limbs are in a completely flaccid and immovable state; it no longer cries; the beating of the heart is scarcely perceptible; the limbs are cold, and deglutition is hardly possible. The infant speedily sinks under this state of weakness, and the post-mortem examination displays a total disorganization of the nervous centre, which explains the symptoms and the death of the infant.

"This softening is more common in the lateral parts of the hemispheres, and near the corpora striata, than in any other part of the brain. The symptoms are severe in proportion as it extends itself, and approaches more nearly to the medulla oblongata. The prognosis is in the last degree unfavorable, for death appears to me to be its inevitable result.

"Such are the lesions which may result from the degrees and varieties of cerebral congestion in new-born infants. The symptoms are generally characterized by a state of depression and prostration; by venous congestion of the integuments of the limbs, trunk, and face; and, above all, by the peculiar signs of pulmonary congestion, which almost always accompanies that of the brain. It is difficult in young infants to observe the effects of apoplexy of one hemisphere upon the opposite side; for, as I have said, in speaking of the development of the brain, this organ, at the time of birth, is but in the germ; it possesses as yet neither the organic forms, nor the vital properties which it acquires in the progress of its development." (P. 623.)

The following passage is also well entitled to a careful consideration.

"*Affections of the Stomach, with or without Obstruction of its Functions.* If I took the symptoms of diseases as the basis of my divisions, I should here be obliged to unite diseases the seat of which would have no mutual relation; for the various affections with which the stomach may be affected give rise to symptoms so different, that it is sometimes very difficult to class them together. But, in dividing the diseases which I have taken upon me to describe according to the anatomy which determines them, I am naturally led to give a complete pathology of the stomach. I subdivide the diseases of the stomach which are developed after birth into passive congestions and inflammations.

"1. *Congestions of the Stomach.* We have seen that, at the time of birth, the stomach is almost always injected. Whatever may be the condition of the general or pulmonary circulation, the abdominal vessels are gorged with black and fluid blood, which flows back towards the capillaries, the multiplied branches of which are injected and obstructed, and give to the coats of the stomach an aspect more or less ruddy. When you open the bodies of these children, you find, on the internal surface of the mucous membrane a ramifying capillary injection, or an injection characterized by its bluish aspect; the colour more decided in the lower parts of the organ; absence of tumefaction, with pulpiness of the mucous tissue; and especially general congestion of the large venous trunks of the abdomen, liver, spleen, venæ cavæ, heart, and lungs. The blood which stagnates in the vessels of the stomach soaks through its layers, mechanically penetrates into the cellular tissue, infiltrates the mucous membrane itself, and is found exuded on the surface of that membrane, so as to colour it with more or less intensity, or even to form a real passive hemorrhage.

"Examples of passive congestion of the stomach in infants at the breast are so numerous, that I could cite a great number. I shall confine myself to the history of a case of congestion of the stomach, which exhibits all the anatomical characters I have just enumerated.

"CASE. Auguste Bourbon, six days old, was brought into the hospital, on the 2d of May. His nurse brought him to the Infirmary, stating that the child was often on the point of suffocation, refused to suck, and slept very little. It was of rather a strong constitution, but its face was puffed, and its limbs livid and œdematous; its respiration difficult, its cry stifled and peculiar; the pulse was imperceptible; the pulsations of the heart were feeble and irregular, and rose at most to fifty in a minute: they were so faint, that they were counted with difficulty. The child was wrapped in flannel; frictions with treacle-water were applied to the body and limbs.

"May 3d. The upper lip was considerably swelled; the child vomited bloody matter. A wash of acidulated decoction of cinchona was ordered.

"4th. The debility increased, vomiting of sanguineous matter continued, and in the evening the patient died.

"The following day a post-mortem examination furnished these appearances: Tumefaction and livid redness of the upper lip; the mucous membrane lining the cheeks was of a livid red; the tongue tumefied, and somewhat inclined to ecchymosis at the root; the œsophagus was much injected; the whole stomach of a livid red; the coats were soft, and easily separated, and also infiltrated with blackish blood. The stomach contained a large quantity of matter, apparently mucous, and of a brown sanguineous appearance, exactly resembling that which the child had vomited. The liver was gorged with blood of an intensely red colour; on its surface appeared a kind of sanguineous dew. Pale fluid blood was effused in the abdominal cavity. The lungs on the left side were crepitant, and filled with air; the lungs on the right side presented appearances the reverse of these, and there was blood effused in the pleura. The heart and the large vessels were very much obstructed, and their tunics were as if soaked in blood; this fluid was also effused in the pericardium. The vessels of the meninges and the surface of the brain were found considerably injected, as also the plexus choroides. The cerebral pulp was of a deep red."

M. Billard remarks, that the condition described is one of real passive congestion, and of very common occurrence at this period of life; and presumes, that the sanguine congestion of the stomach is not always accompanied with a corresponding condition of the contents of the chest; although he believes that abdominal congestion is dependent on the difficult or incomplete establishment of respiration or circulation, which is also a common phenomenon in the early days of existence.

Nothing can exceed the accuracy and patient industry with which the observations are made and recorded. Of course we shall be acquitted of a want of just appreciation of such contributions to our knowledge; they form an integral part of a system, and are indispensable to its perfection. Pathology has of late years received great and unprecedented attention, and its cultivation has received a corresponding impulse. Deprecating misconstruction by its enthusiastic devotees, we may be permitted to doubt the extent of the utility of exclusive attention to the structural changes effected by disease. The conviction that improvements in the treatment of disease are owing less to morbid anatomy than to a careful and philosophic analysis of symptoms, is now indelibly impressed upon those whose opportunities justify our confidence. The most valuable knowledge in prognosis and treatment has ever been, and from its nature always must be, derived from the observation of symptoms and the operation of remedies. This knowledge is stigmatized as empiricism by those who vainly hope that the study of abnormal anatomy can reduce the practice of physic to the certainty of the exact sciences. We are willing to admit the great probability (perhaps absolute certainty) that maladies referred to functional derangement are necessarily occasioned by some deviation from healthy structure, generally designated as *disease*, in contradistinction to *disorder*. This admission will avail but little to our objectors; for if these changes, the vast majority of which are not appreciable, could be detected with certainty, we should not thereby be advanced a line in the improvement of their treatment. The action of a disturbing cause may have for its effect a morbid change of structure; this in its turn may become the cause of functional derangement, which ultimately may be the cause of death. It is here evident that the cause producing the change of structure may be called primary; while the effect of the disturbing action, the change of structure itself, may be regarded as an intermediate, or second cause, which may be an efficient but not a necessary cause of death. Now, where it is only an efficient cause of death, the original cause cannot be found on dissection.

In the majority of mortal diseases the structural change is not the original cause, and we insist that a drawback on the value of morbid anatomy, as estimated by some of its worshippers, is its inadequacy to shew that condition which alone it is useful to know, viz. the state of things before the structural change be wrought, instead of exhibiting results which to know, furnishes but rarely, and in a very moderate degree, information how the evil might be averted. Often the post-mortem appearances seem to indicate, and would certainly influence, if they could previously be foretold, a mode of treatment known by experience to be hurtful: delirium tremens, and the state of the abdomen after lithotomy, bronchitis, adynamic fevers, and some types of puerperal fever, are cases in point. Even the state of an organ taken *per se* would sometimes suggest a mode of treatment, the adoption of which had actually produced death. If, in practice, the etiology of disease, the effects of the influence of remedies, and the minor knowledge deduced from morbid dissections, were applied in conjunction, the art would be increased in usefulness, as it would be elevated in philosophy. Modes of successful practice cannot be deduced from evidences furnished by the state of the organs, excepting in a very few cases; and these become more rare in proportion to their severity: witness phthisis pulmonalis, hydrophobia, cancer, erysipelas, melanosis, fungus hæmatodes, tubercles, scrofula, and others. Moreover, the maladies in which change of structure is not so manifest, or perhaps not even apparent, form a good portion of our heritage of ills; and these will be in a degree neglected, if we rest our hopes of instruction on so insufficient a foundation.

In truth, there is in medicine, as in manners and morality, a fashion, and morbid anatomy is now the mode; for the predilection we are indebted to the French, and we only complain of its too exclusive cultivation. Justice compels the admission, that our scientific obligations to our neighbours are too great to be speedily repaid: less national critics might doubt the possibility of redemption, yet we do not despair, if a generous rivalry be substituted for too close an imitation.

Again we disclaim anything disapprobatory of M. Billard's work, which we cordially commend, advising its immediate translation by any one disposed to a profitable and honourable employment.

